# Low-cost social robot designs for education: a review

**DOI:** 10.3389/frobt.2026.1827676

**Published:** 2026-06-30

**Authors:** Edger P. Rutatola, Frank C. Msonge, Remko Proesmans, Koen Stroeken, Tony Belpaeme

**Affiliations:** 1 IDLab-AIRO, Ghent University—imec, Ghent, Belgium; 2 Department of Computing Science Studies, Faculty of Science and Technology, Mzumbe University, Morogoro, Tanzania; 3 Centre for Anthropological Research on Affect and Materiality (CARAM), Ghent University, Ghent, Belgium

**Keywords:** custom robots, educational robots, human-robot interaction, low-cost robots, robot design, sensors and actuators, social robots, systematic review

## Abstract

Social robots have shown promising potential in educational contexts worldwide, with studies reporting significant cognitive and affective gains when such robots are deployed. However, among other factors, the high cost of commercial robots limits this line of research to a small number of laboratories and hinders large-scale adoption in real-world educational settings, with most studies remaining short-term pilot interventions. Although several reviews exist in this domain, they primarily focus on applications, impacts, and trends, with limited attention to robot design in relation to affordability. Using a systematic literature review, this study identifies the features and capabilities commonly incorporated in low-cost social robots in educational contexts, as well as the design choices that balance effectiveness and affordability. Thirty-four (34) studies describing low-cost custom social robot designs were analysed following PRISMA guidelines. Articles were retrieved from Web of Science, Scopus, IEEE Xplore, and the ACM Digital Library, and analysed using content analysis as well as descriptive and inferential statistics. The findings show that speech-based interaction (73.5%), vision capabilities (53%), and body-part movement (47%) are the most frequently reported features supporting learner–robot interaction. Additional features include expressive displays, touch input, and mobility. While 3D-printed housings are the most commonly adopted solution, alternative low-cost materials such as plywood and plush toys are also used. The use of smartphones as integrated computational and interaction platforms emerged as an innovative approach. Furthermore, commonly used sensors, actuators, and compute platforms are identified. This review provides a design-oriented perspective on developing effective yet affordable social robots and highlights practical design trade-offs for researchers, particularly in low-resource contexts.

## 1 Introduction

A synthesis by Mahdi et al. (2022) identifies several defining qualities of social robots, including being socially evocative, socially situated, sociable, socially intelligent, and socially interactive—that is, the ability to engage users, perceive and respond to social cues, and support meaningful human–robot interaction. In this study, robots exhibiting one or more of these qualities are considered social robots. Such systems are increasingly regarded in educational settings as tools to enhance teaching and learning through social interactions. A meta review by Barakova et al. (2023) highlights that several review studies agree that social robots are beneficial as they can provide personalised learning, support social interactions between learners, nurture creativity through their versatile interaction abilities, kindle positive affect, provide prompt feedback, and motivate learners through their novelty. Empirical studies with social robots playing a central role in the learning process report positive cognitive and affective impacts on learners (Belpaeme et al., 2018), solidifying their potential.

Reflecting these capabilities and benefits, a variety of social robots are available on the market. However, in educational contexts, the humanoid NAO robot by Aldebaran Robotics is the most widely used (Belpaeme et al., 2018; Gardenghi and Gherardi, 2024). While hundreds of other platforms have been reported (Mahdi et al., 2022), notable examples include Keepon, Tega, Dragonbot, iRobiQ, and Robovie (Belpaeme et al., 2018; van den Berghe et al., 2019).

While research on social robots for education has progressed for more than two decades, their sustained, full-time adoption in classrooms and comparable real-world settings remains limited (Barakova et al., 2023). Next to technical and interaction-related challenges encountered by robots in real-world settings, the high cost of robots is frequently cited as a major barrier to accessibility and scalability (Barakova et al., 2023; Souza et al., 2024; Zakipour et al., 2016). Consequently, such research remains concentrated in a limited number of research laboratories worldwide (Lampropoulos, 2025), and most field studies remain limited in scope and duration.

In Sub-Saharan Africa, research on social robots for education is relatively recent and remains under-explored. For instance, two field-based studies conducted in the United Republic of Tanzania report both cognitive and affective gains (Ntahomvukye et al., 2025), as well as teachers’ cautious yet positive attitudes toward the use of robots in schools (Rutatola et al., 2025). Nevertheless, despite this potential, large-scale adoption for now remains unrealistic, particularly in light of the country’s more than 18,055 government-run primary schools (National Bureau of Statistics, 2024). Consequently, the development of low-cost social robotic platforms is essential for enabling scalable and sustainable deployment in this and comparable educational contexts.

One key factor underlying these limitations is the cost of commercial social robots, which tends to escalate due to their comprehensive designs that integrate a wide range of sensors and actuators to support customisation across diverse use cases. In response, researchers worldwide often develop bespoke social robots tailored to specific research objectives, incorporating only the components necessary to support the intended tasks and interactions. The availability of low-cost fabrication methods, such as 3D printing (Maure and Bruno, 2023; Narejo et al., 2024; Sanchez-Orozco et al., 2025), the use of plywood (Petrovič and Kostrian, 2024), and other affordable innovations, has further enabled the integration and housing of components and the realisation of complete robot housings.

The adjective “low-cost” is commonly used to describe various technological approaches and artifacts. A study by Salmerón-manzano and Manzano-agugliaro (2020) stresses that there is no general agreement on the definition of low-cost technology. They present one view that the term might refer to a technology that, in comparison, offers a more economical solution than that of similar commercial solutions. It describes solutions that use more accessible technologies to achieve similar results as those of advanced technologies. Without defining a specific price threshold, given the contextual and application-dependent nature of affordability, this line of thinking is adopted in this study, where “low-cost” social robots refer to robots whose designs leverage accessible technologies to economically achieve functionalities and results comparable to those of their commercial counterparts.

Therefore, recognising the need for low-cost social robots and the diversity of available design approaches, this study aims to systematically review existing research that reports on the design and development of low-cost custom social robots. Specifically, it seeks to address the following research questions.

- RQ1: Which robot features and capabilities are most commonly incorporated in low-cost social robot designs for education?
- RQ2: What tools, materials, and design approaches are commonly employed to ensure that custom robots for education remain low-cost?

By addressing these questions, this review aims to inform researchers about practical strategies for designing and building custom social robots with a specific focus on low-resource settings, thereby contributing to the democratisation of robotics research and deployment.

## 2 Contributions of the study

The use of social robots in education is a well-established topic in Human-Robot Interaction (HRI) research, and several reviews and meta-analyses have synthesised the field by surveying the different pedagogical interactions afforded by robots, the educational outcomes, and recommendations for design and deployment (Belpaeme et al., 2018; Woo et al., 2021; Lampropoulos, 2025; Barakova et al., 2023). While some reviews provide broad overviews, others are more specific, examining the use of social robots for education and interaction in a subgroup of users in depth. For example, reviews by Kouroupa et al. (2022) and Papakostas et al. (2021) focus on users in the special education category. Most of these reviews focus on learner–robot interactions (LRIs) and their outcomes, but less attention has been paid to *how these robots are built*, which technical design choices enable interaction, and how such choices relate to affordability, accessibility, and scalability. Existing reviews focus on the LRIs without explicit consideration of the type of robot used: commercial or custom, nor the affordability and accessibility of that robot.

More technically inclined, a study by Evripidou et al. (2020) reviews the various available educational robotics platforms. They categorise the platforms based on the skills needed by students to program the robots. Moreover, they highlight expected learning outcomes when educational robots are used in the learning process. However, the roles of the robots described are as programmable platforms for students to learn Science, Technology, Engineering, and Mathematics (STEM) concepts, and not to enhance the learning process through social interactions. More closely aligned with the focus of the present study, a review by Manna et al. (2024) provides a qualitative examination of the technical features and educational impact of a range of robots used in STEM education. It evaluates robots with socially interactive features, citing their hardware components (compute, sensors, and actuators) and indicative prices. However, beyond listing technical components, it does not provide a comprehensive analysis of robots’ features related to appearance, functional capabilities, and underlying design approaches, all of which are critical considerations when affordability and scalability are primary design goals. A further relevant study is the comprehensive review by Mahdi et al. (2022), which synthesises the design and evolution of 344 social robots reported in studies published prior to November 2020. The review provides detailed information on robot features, embodiment, and application contexts across domains, including education. However, its primary focus is on broad characterisation of social robots rather than affordability or cost-constrained design. As such, additional interpretation is required when using its findings to inform the development of low-cost social robots, particularly in resource-constrained educational settings.

This review builds on and extends these earlier studies in several important ways. First, whereas prior reviews primarily focus on interaction outcomes, we take the robot itself as the unit of study, and provide a systematic and technically grounded synthesis of the design features such as hardware, appearance, and capabilities that enable LRIs. Second, we focus specifically on custom low-cost social robots, a segment of the literature that remains underexplored despite its importance for accessibility, scalability, and deployment in resource-constrained educational settings. In doing so, our review complements existing interaction- and outcome-focused reviews by offering actionable insights for researchers aiming to design and build their own low-cost social robots for educational contexts.

## 3 Materials and methods

### 3.1 Research design

Given that the objective of this study is to examine the design features and capabilities of existing low-cost social robots, a literature review-based approach was deemed the most appropriate methodology. To minimise bias and enhance transparency and reproducibility, we therefore conducted a systematic literature review. We acknowledge that social robot designs exist that are not reported in scholarly publications; however, their inclusion would reduce reproducibility and introduce additional methodological complexity. We also acknowledge that commercially available social robots often stem from research and are used in academic studies; although relevant, they are not consistently accompanied by sufficient hardware and software documentation to enable systematic comparison across studies. Consequently, this study focuses on robot designs reported in peer-reviewed publications, as these provide a traceable and amenable source of design information within the scope of a systematic review.

### 3.2 Database search

We ran our search across four databases: Web of Science (WoS), Scopus, IEEE Xplore, and the ACM Digital Library. Web of Science and Scopus were selected because they contain papers more focused towards applications and user studies, thus informing RQ1. IEEE Xplore and ACM Digital Library contain relatively more technical papers, addressing RQ2. In WoS, Scopus, and IEEE Xplore, a metadata-level search was conducted. However, the metadata search in the ACM Digital Library retrieved a large number of irrelevant results; therefore, the search was restricted to abstracts only. We customised a search query for each database (see Appendix 1). Essentially, articles retrieved had to satisfy the following Boolean condition:

(“social robot*” OR “humanoid robot*” OR “anthropomorph*” OR “human-like robot*”) AND (“design*” OR “feature*” OR “appearance” OR “embodiment”) AND (“education*” OR “classroom” OR “teach*” OR “learner*” OR “tutor*” OR “peer”) AND (“affordab*” OR “low-cost” OR “cost-effective*” OR “open source” OR “open-source” OR “open hardware” OR “DIY” OR “Arduino” OR “Raspberry Pi”) AND “robot*” AND NOT (“industrial robot*” OR “manufacturing robot*”).

We excluded industrial and manufacturing robots to ensure that we only retrieved studies involving social robots. Moreover, to be able to establish a trend, we searched for articles from 2015 to 2025, with the cut-off on 6 October 2025.

### 3.3 Quality control

We followed the Preferred Reporting Items for Systematic Reviews and Meta-Analyses (PRISMA) 2020 guidelines to ensure rigour and reproducibility (Page et al., 2021). Through a Python script, the titles and Digital Object Identifiers (DOIs) of all the retrieved articles were scrutinised to remove duplicates. Thereafter, the unique articles were subjected to title and abstract-level screening for relevance. The following exclusion criteria were agreed upon prior to the screening process.

- If the article’s language is not English
- If the article reports on an isolated robot component (e.g., arms, eyes, skin, *etc.*)
- If the reported research uses a commercial robot
- If the research does not include a physical robot
- If the research does not focus on robot design
- If the research does not present an original robot design contribution (e.g., review studies, studies that primarily report the use of existing robots without sufficient technical detail on their hardware and/or software design, or studies describing robots whose primary design specifications are reported in another publication)
- If the robot is used as a tool: if the robot is used primarily as a technical learning or programming platform, where any interactive capabilities (e.g., speech output or responses) are not designed for social engagement but serve only as programmable outputs for educational or task-based purposes. In such cases, interaction is possible, but the system is not designed with social interaction as its primary goal.

**FIGURE 1:**
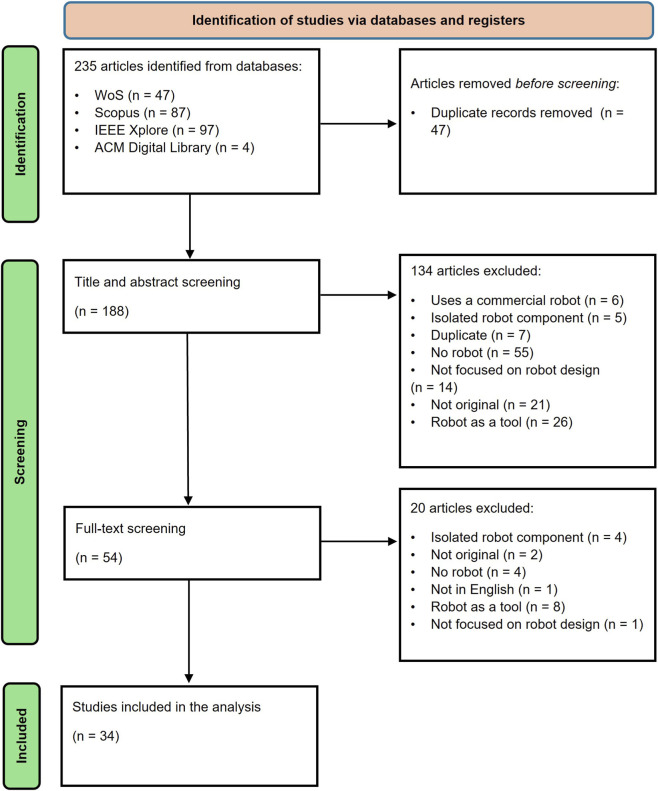
PRISMA flowchart (Page et al., 2021): 235 articles were retrieved from the databases, 54 full texts were screened, and ultimately, only 34 articles were analysed in this review.

Furthermore, it is important to note that by adapting the philosophy that cost and affordability are relative (Salmerón-manzano and Manzano-agugliaro, 2020), we did not specify an objective cost threshold as an inclusion or exclusion criterion. Alternatively, as indicated in the search query, we relied on the respective author(s)’ self-declaration that their robot design is low-cost/affordable. This approach allowed the inclusion of a wider array of design options from different resource and application contexts. The resulting subset was fully reviewed, and the results are reported in Section 4. The PRISMA diagram showing the number of articles excluded based on each criterion is presented in Figure 1.

Abstract screening and full-text reviews were conducted independently by two reviewers. Any discrepancies regarding article inclusion were resolved through discussion until consensus was reached. For full-text data extraction, each reviewer used a separate structured extraction matrix coded in a spreadsheet. Inter-rater reliability was assessed on a random 15% subsample based on agreement across the extracted and coded features, yielding a Cohen’s kappa of 0.793 (96.15% agreement), indicating substantial agreement. Any discrepancies in the extracted data were subsequently discussed until consensus was reached.

### 3.4 Analysis

The articles were analysed thematically according to the following predefined themes: (1) motivations for designing custom robots, (2) robot appearance, (3) robot features and capabilities, (4) tools and components used, (5) design approaches, (6) demonstrated applications beyond laboratory testing, (7) software and design availability, and (8) estimated prototype costs. To provide additional context, we also analysed information such as authors’ affiliations, study locations, and publication types. Depending on the nature of the extracted data, we applied either descriptive statistics (for quantitative variables) or content analysis (for qualitative data). Spreadsheets and coding matrices were used to streamline the analysis. In addition, inferential statistical analyses were conducted to examine potential associations between prototype cost and selected design and contextual variables.

## 4 Results

A total of 235 articles were retrieved from the four databases: 47 from WoS, 87 from Scopus, 97 from IEEE Xplore, and 4 from the ACM Digital Library. After screening for duplicates, 188 articles were retained. Two levels of screening were done: (1) title and abstract screening, in which 134 articles were excluded following the criteria described in Section 3.3 and (2) full-text screening of the subset, in which 20 articles were further excluded following the same criteria. At the end, only 34 articles were deemed relevant for our review. Figure 1 details the process.

### 4.1 Metadata analysis and contextual overview

#### 4.1.1 Publication types

Most of the reviewed studies were conference publications, accounting for 62% (n = 21) , while 38% (n = 13) were journal publications. This shows that most researchers prefer to present their work at conferences, where prototype designs and early-stage experiments are often shared. Moreover, most technical conferences have design tracks and competitions in which most such works are presented. While each article was presented at a unique conference, certain terms appeared frequently in the conference titles. The most common were robot(ics) (n = 10) , interact(ion) (n = 5) , human (n = 5) , technology (n = 4) , and systems (n = 3) . This pattern suggests that the majority of authors primarily target technically oriented conferences, while still maintaining a notable emphasis on human-centred research.

As for the 13 journal articles, two articles were published in the IEEE Access journal, while the rest appeared in distinct journals. Analysis of journal names shows that the most recurring themes are engineering (n = 3) , technology (n = 2) , and robot(ics) (n = 2) .

#### 4.1.2 Keywords and index terms

**FIGURE 2:**
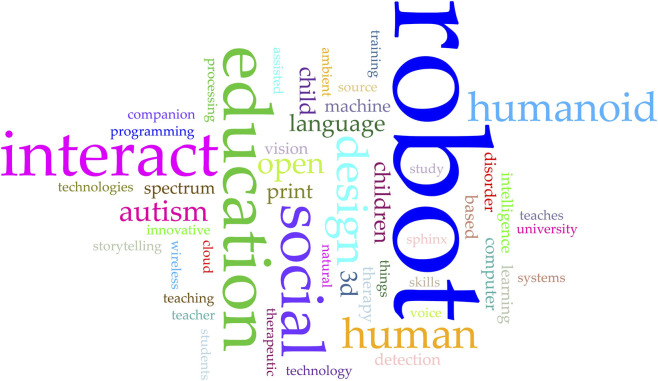
Visualisation of frequent words in the articles’ keywords and index terms reveals that variations of the words robot, interact, education, social, and design were the most common.

For this analysis, we first selectively normalised relevant words in the keywords and index terms by converting them to their lemmas, while other terms were left unchanged. The resulting dataset contained a total of 325 words and 163 unique word forms. As expected, the word “robot” (originally robot(s) or robotic(s)) dominated the keywords and index terms in the publications (15.4%, n = 50 ). Interestingly, “interact” (originally interaction or interactive) emerged as the second most prevalent term (4.6%, n = 15 ), suggesting that interaction was a central objective in many of the robot designs. Similarly, “education” also appeared frequently (4.6%, n = 15 ). Other notable common words included social (4%, n = 13 ) and design (2.5%, n = 8 ). Figure 2 visualises the occurrences.

#### 4.1.3 Authors’ affiliations

**FIGURE 3:**
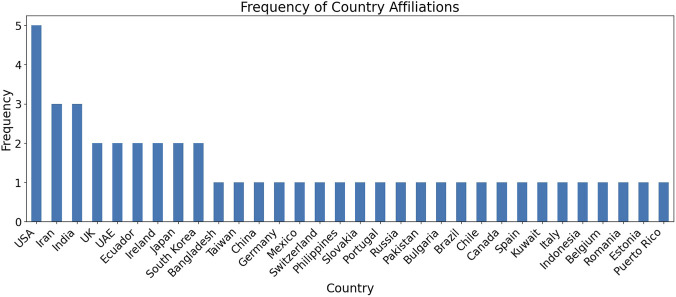
Most authors were affiliated with institutions in the USA, followed by Iran and India.

We analysed author affiliations to gauge the geographical distribution of custom robot development initiatives (production). Each publication was treated as an independent unit of analysis. If more than one author in a publication was affiliated with the same country, the country was only counted once for that publication. As a result, our analysis yielded 46 country occurrences across the included publications. As depicted in Figure 3, the United States of America (USA) appeared the most (approx. 11%, n = 5 ). Iran and India both appeared 6.5% (n = 3) of the time. The United Kingdom (UK), the United Arab Emirates (UAE), South Korea, Japan, Ireland, and Ecuador had a share of approximately 5.9% (n = 2) each.

#### 4.1.4 Study area

In addition, we analysed study areas to better understand where actual interventions take place, beyond just laboratory-based development. In the majority of the reviewed studies, the country in which the research was conducted was explicitly stated, irrespective of whether field experiments were performed. For cases in which the study location was not clearly specified, the authors’ institutional affiliations were used as a proxy when all authors shared the same affiliation. Using this approach, we were able to identify the study location for all but 14.7% (n=5) of the articles.

**FIGURE 4:**
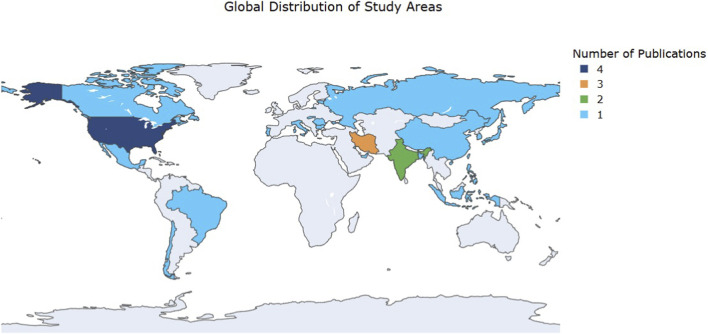
The USA had the most studies, followed by Iran and India. Studies were conducted in Asia, Europe, North America, and South America.

As shown in Figure 4, the United States accounted for the largest number of studies, followed by Iran and India. Further analysis based on continent reveals that most studies (48%) were conducted in Asia, followed by 26% in Europe, 13% in North America, 8.7% in South America, and 4.3% in Eurasia (Europe/Asia). None of the studies were conducted in Africa, Oceania, or Antarctica.

### 4.2 Motivations for designing low-cost custom robots

The most cited motivation for embarking on low-cost custom robot designs was the unaffordability of commercially available robots, hindering extensive research and adoption (Polyntsev et al., 2021; Zakipour et al., 2016; Ivanova et al., 2025; Shayan et al., 2016; Nagasawa and Yoshikubo, 2024; Singh et al., 2023; Souza et al., 2024). This was true even for institutions in the Global North (Polyntsev et al., 2021; Nagasawa and Yoshikubo, 2024). The study by Wu et al. (2016) details the costs of some commercial social robots based on their features, ranging from a few thousand USD to 1 million. Another motivation was a need for a robot tailored to a specific use case. For example, Shayan et al. (2016) specifically designed a culturally grounded social robot suitable for the Middle East.

### 4.3 Robot appearance

#### 4.3.1 Robot morphology

Various robot morphologies have been adopted in the reviewed studies. We have grouped them into three categories: anthropomorphic (full body or partial), referring to robots whose appearances are human-like; zoomorphic, referring to robots whose appearances are inspired by non-human animals; and others, containing robots whose appearances fall under neither of the former categories. The majority of robots were anthropomorphic (67.6%, n = 23 ), implying that human-like robots were mostly preferred in an education context. Zoomorphic robots accounted for 14.7% (n = 5) , while 17.6% (n=6) were classified as others. Table 1 lists all the reviewed robots and their respective morphologies.

**TABLE 1:** Robot morphology.

S/N	Robot name	Morphology	Articles
1	RoboParrot 2.0	Zoomorphic	Shayan et al. (2016)
2	ProbogotchiI	Zoomorphic	Simut et al. (2016)
3	Meow	Zoomorphic	Polyntsev et al. (2021)
4	Cloud Sphinx	Zoomorphic	Ma et al. (2020)
5	Anita V4	Zoomorphic	Budiharto (2023)
6	Tiny	Anthropomorphic - full body	Jobaida et al. (2024)
7	Sanpo	Anthropomorphic - full body	Lin (2022)
8	Robby	Anthropomorphic - full body	Rodríguez-Calderón and Belmonte-Izquierdo (2022)
9	PixelBot	Anthropomorphic - full body	Maure and Bruno (2023)
10	Otisma	Anthropomorphic - full body	Bouhali et al. (2023)
11	Mirrly	Anthropomorphic - full body	Yamini et al. (2024)
12	Lilli	Anthropomorphic - full body	Petrovič and Kostrian (2024)
13	HBS-1	Anthropomorphic - full body	Wu et al. (2016)
14	—	Anthropomorphic - full body	Malarvizhi et al. (2024)
15	—	Anthropomorphic - full body	Al Omoush et al. (2024)
16	Yaren	Anthropomorphic - partial	Sanchez-Orozco et al. (2025)
17	Woody	Anthropomorphic - partial	Hayosh et al. (2020)
18	Tinku	Anthropomorphic - partial	Singh et al. (2023)
19	Shybo	Anthropomorphic - partial	Lupetti (2017)
20	Roboldo	Anthropomorphic - partial	Souza et al. (2024)
21	RASA	Anthropomorphic - partial	Zakipour et al. (2016)
22	JF-2 and JF-mini	Anthropomorphic - partial	Kim et al. (2024)
23	Flexi	Anthropomorphic - partial	Alves-Oliveira et al. (2022)
24	ERO	Anthropomorphic - partial	Raj et al. (2023)
25	Arash	Anthropomorphic - partial	Meghdari et al. (2018)
26	—	Anthropomorphic - partial	Narejo et al. (2024)
27	—	Anthropomorphic - partial	Nagasawa and Yoshikubo (2024)
28	—	Anthropomorphic - partial	Ivanova et al. (2025)
29	Robotic Study Companion (RSC)	Others	Baksh et al. (2024)
30	Pudu	Others	Ruiz-del Solar et al. (2021)
31	GeeBot	Others	Simao et al. (2018)
32	Atent@	Others	Berrezueta-Guzman et al. (2021)
33	—	Others	Galvez et al. (2022)
34	—	Others	Diano and Claveau (2015)

A further analysis of the anthropomorphic robots revealed that 43.5% (n = 10) were full-body humanoids (see Table 1). The remainder, 56.5% (n = 13) , were only partially anthropomorphic, as described in Table 2. As for the zoomorphic robots, 3 out of 5 had a cat-like appearance. One was designed to look like a caged macaw parrot, and the other was a stuffed animal that does not resemble a specific animal. Table 3 provides more details. Moreover, for the “others” category, the majority (five out of six) were display screens (2 of which were smartphones) on either mobile or immobile bases. This stresses the importance of display in human-robot interaction. The other was an egg-shaped robot with eyes. Table 4 provides more details.

**TABLE 2:** Partially anthropomorphic.

Features	Frequency	Articles
Eye, nose, and mouth	1	Lupetti (2017)
Head and torso (with or without a base)	3	Raj et al. (2023); Ivanova et al. (2025); Alves-Oliveira et al. (2022)
Torso, arms, and wheels	1	Nagasawa and Yoshikubo (2024)
Head, torso, and arms	4	Sanchez-Orozco et al. (2025); Souza et al. (2024); Zakipour et al. (2016); Hayosh et al. (2020)
Head, torso, arms, and wheels (with or without a waist)	4	Narejo et al. (2024); Meghdari et al. (2018); Singh et al. (2023); Kim et al. (2024)
**Total**	**13**	

**TABLE 3:** Zoomorphic.

Resembling animal	Frequency	Articles
Cat	3	Ma et al. (2020); Polyntsev et al. (2021); Budiharto (2023)
Macaw Parrot	1	Shayan et al. (2016)
Undefined: Stuffed animalistic doll with two legs, two arms, and a long trunk-like nose	1	Simut et al. (2016)
**Total**	**5**	

**TABLE 4:** Other morphologies.

Description	Frequency	Articles
Cube-shaped with a screen, two flippers, and a base	1	Baksh et al. (2024)
A touch screen with a cubic cover and wheels	1	Berrezueta-Guzman et al. (2021)
A tablet (smartphone) mounted on a mobile vertical device	1	Ruiz-del Solar et al. (2021)
Egg-shaped with eyes	1	Simao et al. (2018)
A smartphone on a base with four mini legs	1	Diano and Claveau (2015)
A box with an LCD and a camera mounted on top	1	Galvez et al. (2022)
**Total**	**6**	

#### 4.3.2 Preferred colours

**FIGURE 5:**
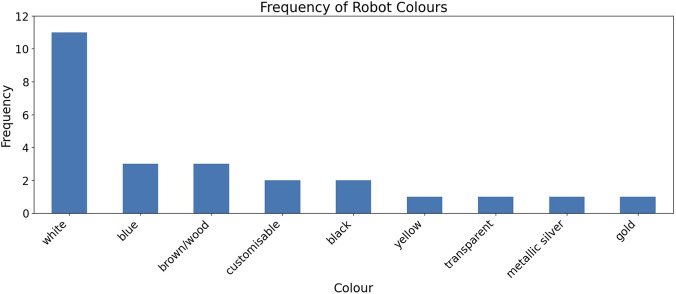
White was the most preferred colour, followed by brown/wood and blue.

In addition to the overall morphology, we also analysed the colours used in the anthropomorphic robots. This was motivated by the study by Barenbrock et al. (2024), which highlighted that a robot’s surface colour influences users’ implicit perception of the robot. In education, the learners’ implicit perception of the facilitator might impact learning. In the reviewed articles, only 8.7% (n = 2) did not state the robot’s colour, or it was not obvious from the included images. For the remainder, as depicted in Figure 5, white was the most preferred colour, whether as the sole colour or in combination with others. Brown/wood and blue colours were also common. For some robots, the colour was completely customisable (Kim et al., 2024; Alves-Oliveira et al., 2022).

Notably, in most of the reported colour combinations, white was included as one of the paired colours, appearing in white–yellow, white–black, and white–blue designs (each occurring once) (Rodríguez-Calderón and Belmonte-Izquierdo, 2022; Narejo et al., 2024; Malarvizhi et al., 2024). Only one robot featured a combination that did not include white (black and gold) (Sanchez-Orozco et al., 2025).

#### 4.3.3 Dimensions

As depicted in Table 5, 29.4% (n = 10) of the studies provided at least one dimension (height, width, and/or length) of their robot. The average dimensions were 63.3 cm ± 46.6 cm, 25.1 cm ± 14.8 cm, and 17.1 cm ± 8.5 cm for the height, width, and length, respectively.

**TABLE 5:** Robot dimensions.

S/N	Robot name	Height (cm)	Width (cm)	Length (cm)	Articles
1	Atent@	9.398	11.176	12.7	Berrezueta-Guzman et al. (2021)
2	MEOW	18	17	14	Polyntsev et al. (2021)
3	Robby	20	20	20	Rodríguez-Calderón and Belmonte-Izquierdo (2022)
4	Roboldo	47	–	–	Souza et al. (2024)
5	YAREN	50	21.5	9	Sanchez-Orozco et al. (2025)
6	–	52.7	17.8	33	Al Omoush et al. (2024)
7	RASA	56	–	–	Zakipour et al. (2016)
8	HBS-1	120	33	14	Wu et al. (2016)
9	Arash	130	55	–	Meghdari et al. (2018)
10	Pudu	130	–	–	Ruiz-del Solar et al. (2021)
	**Mean**	**63.3**	**25.1**	**17.1**	
	**Standard deviation** (σ)	**46.6**	**14.8**	**8.5**	

#### 4.3.4 Housing materials

**FIGURE 6:**
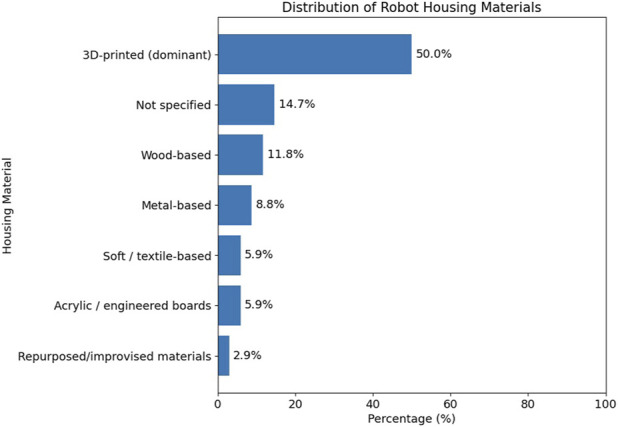
Distribution of robot housing materials. Nearly half of the robots used 3D-printed housing as the dominant structural choice. Other preferred materials, in descending order of frequency, include wood-based, metal-based (e.g., aluminium and steel), acrylic or engineered boards, soft or textile-based materials, and repurposed components.

To achieve low-cost robot housing, 3D printing was the most preferred approach. Half of the studies used 3D-printed housings, whether partially or fully. Where 3D printing was used to only produce parts of the housing, it was supplemented by aluminium alloy (Zakipour et al., 2016), a plush toy (Shayan et al., 2016), steel parts Rodríguez-Calderón and Belmonte-Izquierdo (2022), and metal springs (Nagasawa and Yoshikubo, 2024). Other common materials that formed whole or part of the robots’ housing included (ply-)wood (Hayosh et al., 2020; Diano and Claveau, 2015; Singh et al., 2023; Petrovič and Kostrian, 2024), aluminium alloys (Zakipour et al., 2016; Ruiz-del Solar et al., 2021; Meghdari et al., 2018; Alves-Oliveira et al., 2022), acrylic sheets (Al Omoush et al., 2024; Lin, 2022), and plush toys or felt and cotton (Souza et al., 2024; Simut et al., 2016; Shayan et al., 2016). Figure 6 summarises the prevalence of robot housing materials.

### 4.4 Robot features and capabilities

**FIGURE 7:**
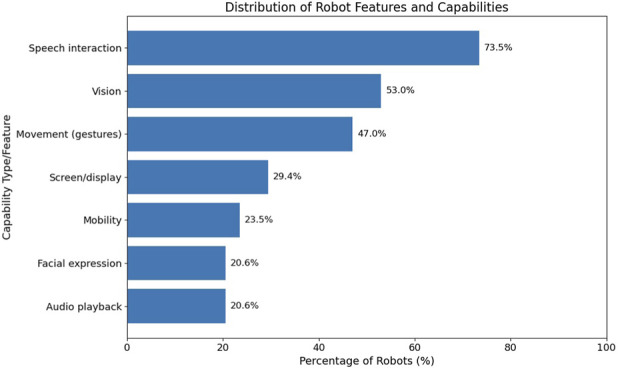
Speech interaction was the most common capability, followed by vision and movement of body parts for non-verbal communication. Other features and capabilities included screen-based display, mobility, facial expressions, and audio playback. It should be noted that these are not mutually exclusive, and individual robots may exhibit multiple features and capabilities.

We further analysed the features and capabilities of the designed robots to understand the commonly incorporated features for interaction. These were detailed in all but one study. As visualised in Figure 7, most robots (73.5%, n = 25 ) are described to interact through speech, having either or both speech recognition and speech synthesis (text-to-speech) capabilities. Moreover, 53% (n = 18) of the robots had vision capabilities. This enabled them to perform tasks such as face, object, colour, shape, and emotion detection/recognition, as well as distance estimation (Singh et al., 2023; Souza et al., 2024; Narejo et al., 2024; Shayan et al., 2016; Ma et al., 2020; Kim et al., 2024; Budiharto, 2023). 47% (n = 16) of the robots can move body parts, such as an arm or head. In addition to enabling them to accomplish certain physical tasks, these movements are crucial for non-verbal communication (Alves-Oliveira et al., 2022; Yamini et al., 2024; Singh et al., 2023). Similarly, 20.6% (n = 7) of the studies report using dynamic facial displays (on screens or projected interfaces) and LEDs to express emotions as another form of non-verbal communication (Souza et al., 2024; Polyntsev et al., 2021; Ivanova et al., 2025; Lupetti, 2017; Maure and Bruno, 2023; Hayosh et al., 2020; Nagasawa and Yoshikubo, 2024).

**FIGURE 8:**
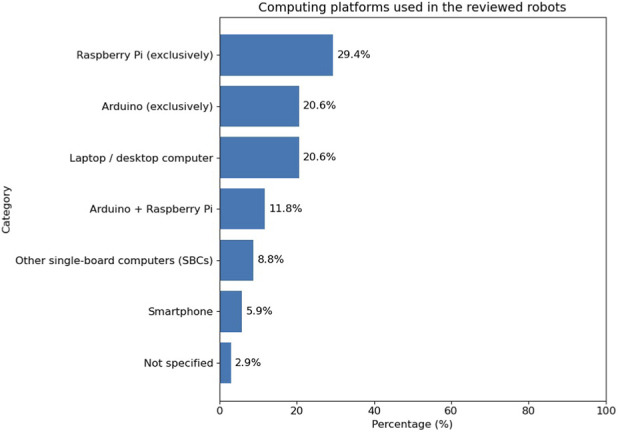
Raspberry Pi single-board computers (SBCs) were the most preferred computing platform, either exclusively or alongside Arduino microcontrollers. Approximately one-fifth of the robots used Arduino as the sole computing platform. External computing resources, including laptops, desktops, and smartphones, were also commonly used.

Beyond these interaction capabilities, 29.4% (n = 10) of the robots were reported to have an attached screen or smartphone through which they could display content. This enhanced the interaction with users, especially in an education setting. The same screens were sometimes used to receive touch input from users as a form of interaction (Kim et al., 2024; Berrezueta-Guzman et al., 2021; Nagasawa and Yoshikubo, 2024; Alves-Oliveira et al., 2022; Yamini et al., 2024). Other ways of receiving touch inputs were through dedicated tactile systems and use of buttons (Simut et al., 2016; Alves-Oliveira et al., 2022; Baksh et al., 2024).

In addition to these interaction mechanisms, locomotion/mobility was highlighted in 23.5% (n = 8) of the studies. This was either achieved autonomously or through teleoperation (Al Omoush et al., 2024; Meghdari et al., 2018; Nagasawa and Yoshikubo, 2024; Diano and Claveau, 2015; Bouhali et al., 2023; Raj et al., 2023; Lin, 2022; Ruiz-del Solar et al., 2021). In addition, 20.6% (n=7) of the robots report the ability to play audio files/sound (Raj et al., 2023; Singh et al., 2023; Baksh et al., 2024; Hayosh et al., 2020; Malarvizhi et al., 2024; Alves-Oliveira et al., 2022; Petrovič and Kostrian, 2024). Lastly, a study by Meghdari et al. (2018) report the ability of sound source localisation.

It is important to note that several of these advanced capabilities, particularly speech and vision processing, often relied on cloud services. Thus, we report the *ability to connect to the internet* as an independent and important feature.

### 4.5 Tools and components used

#### 4.5.1 Compute

All but one study specified the computing platform used by their robot. About 30.3% (n=10) relied exclusively on Raspberry Pi as the main processing unit. 21.2% (n = 7) of the studies relied exclusively on Arduino microcontrollers. Four studies (12.1%) used both Raspberry Pi and Arduino. In such studies, the Arduino handled low-level control of sensors and actuators, enabling functions such as mobility, LED control, motor movement, and environmental sensing. On the other hand, the Raspberry Pi single-board computer (SBC) served as the main processor, managing more resource-intensive tasks such as AI and deep-learning functions (e.g., face, image, voice, and emotion recognition), cloud connectivity, and graphical user interfaces (GUIs) (Ma et al., 2020; Narejo et al., 2024; Al Omoush et al., 2024; Raj et al., 2023).

While Raspberry Pi was the most popular SBC, Latte Panda, NVIDIA Jetson Nano, and NXP i.MX6-based board were each used by one study (Sanchez-Orozco et al., 2025; Bouhali et al., 2023; Singh et al., 2023). Other robots (n = 7) used an external laptop or desktop computer for processing (Kim et al., 2024; Zakipour et al., 2016; Wu et al., 2016; Meghdari et al., 2018; Alves-Oliveira et al., 2022; Simut et al., 2016; Shayan et al., 2016). Lastly, 2 robots used smartphones as their main processing units (Nagasawa and Yoshikubo, 2024; Diano and Claveau, 2015). Figure 8 summarises the computing platforms used in the reviewed robots.

#### 4.5.2 Sensors

We further analysed the sensors used by the robots to realise the features and capabilities described in Section 4.4. The majority (70.6%, n = 24 ) of the robots are reported to include cameras for vision capabilities. Depending on the requirement, either 2D (RGB) or 3D (RGB-D) cameras were used (Petrovič and Kostrian, 2024; Kim et al., 2024; Ruiz-del Solar et al., 2021; Sanchez-Orozco et al., 2025). The number of cameras mounted also varies depending on the study requirements.

In addition to vision sensors, microphones were also among the commonly used sensors (44.1%, n=15 ), specifically for audio input. Some studies, such as those by Budiharto (2023) and Baksh et al. (2024) provide specifications of the microphones they use. As for the robots with obstacle detection capabilities, either or both ultrasonic and infrared sensors were said to be used, with others mentioning proximity, sonar, and time-of-flight (ToF) sensors (Yamini et al., 2024; Lin, 2022; Narejo et al., 2024; Meghdari et al., 2018; Singh et al., 2023; Simao et al., 2018; Berrezueta-Guzman et al., 2021; Nagasawa and Yoshikubo, 2024; Jobaida et al., 2024).

Beyond these sensing modalities, several studies report using UM7-LT, MPU6050 6-axis IMU, gyroscopes, and accelerometers for balance and orientation (Wu et al., 2016; Petrovič and Kostrian, 2024; Meghdari et al., 2018; Zakipour et al., 2016; Diano and Claveau, 2015). Accelerometers were also used to detect movement intensity and frequency (Zakipour et al., 2016; Berrezueta-Guzman et al., 2021). Regarding angle and directional input, X-box 360 joysticks, motor encoders, and flex sensors were employed (Ruiz-del Solar et al., 2021; Singh et al., 2023; Lin, 2022; Al Omoush et al., 2024; Meghdari et al., 2018).

Furthermore, a range of other sensors were reported, including LiDAR for navigation (Meghdari et al., 2018; Kim et al., 2024); Force Sensitive Resistors (FSR) and capacitive sensors for touch (Simut et al., 2016; Alves-Oliveira et al., 2022); physical buttons (Maure and Bruno, 2023); and kinematic sensor for gesture recognition (Meghdari et al., 2018). It should also be noted that for robots that incorporated a smartphone or tablet, the sensors contained in the devices were used (Alves-Oliveira et al., 2022; Nagasawa and Yoshikubo, 2024).

#### 4.5.3 Actuators

In addition to sensors, we analysed the actuators employed to provide the aforementioned robot capabilities. Regarding movement, servo motors were widely used, ranging from standard servo motors (Ma et al., 2020; Maure and Bruno, 2023; Narejo et al., 2024) to DC motors (Rodríguez-Calderón and Belmonte-Izquierdo, 2022; Meghdari et al., 2018; Singh et al., 2023; Shayan et al., 2016), as well as Dynamixel motors (Singh et al., 2023; Yamini et al., 2024; Sanchez-Orozco et al., 2025; Alves-Oliveira et al., 2022; Hayosh et al., 2020; Kim et al., 2024). The specifications of the actual motors are often included in the studies.

For audio output, studies report using speakers of varying specifications (Baksh et al., 2024; Lin, 2022; Jobaida et al., 2024), sometimes driven *via* audio amplifiers (Berrezueta-Guzman et al., 2021). Visual output commonly relied on LEDs, either for simple visual cues (Baksh et al., 2024; Shayan et al., 2016) or to display facial expressions and emotions (Souza et al., 2024). Additionally, some robots incorporated microprojectors to render 3D facial expressions (Nagasawa and Yoshikubo, 2024). The specifications of these devices are also included in the studies.

Furthermore, two studies report the use of Shape Memory alloys (SMAs) as artificial muscles in several robots’ body parts (Nagasawa and Yoshikubo, 2024; Wu et al., 2016). It is also worth noting that robot designs containing an embedded smartphone or tablet also used the device’s inbuilt actuators for interactive output (Ruiz-del Solar et al., 2021; Diano and Claveau, 2015). Table 6 provides a summary of the actuators used.

**TABLE 6:** Summary of actuators used.

Actuator type	Purpose/ Function	Articles
DC Motors	General rotational movement	Rodríguez-Calderón and Belmonte-Izquierdo (2022), Meghdari et al. (2018), Singh et al. (2023), Shayan et al. (2016)
Servo Motors	Controlled rotational movement/precise positioning	Ma et al. (2020), Maure and Bruno (2023), Narejo et al. (2024)
Dynamixel Motors	Advanced position, speed, and torque control	Singh et al. (2023), Yamini et al. (2024), Sanchez-Orozco et al. (2025), Alves-Oliveira et al. (2022), Hayosh et al. (2020), Kim et al. (2024)
Shape Memory Alloys (SMAs)	Artificial muscles/fine movements	Nagasawa and Yoshikubo (2024), Wu et al. (2016)
Speakers	Audio output	Baksh et al., 2024; Lin (2022), Jobaida et al. (2024), Berrezueta-Guzman et al. (2021)
LEDs	Visual output (Simple cues or expressive displays (faces, emotions))	Baksh et al. (2024), Shayan et al. (2016), Souza et al. (2024)
Microprojectors	Visual output (Render 3D facial expressions)	Nagasawa and Yoshikubo (2024)
Embedded smartphone/tablet actuators	Interactive output (built-in actuators: speakers, screens, *etc.*)	Ruiz-del Solar et al. (2021), Diano and Claveau (2015)

### 4.6 Design approach

Most of the studies (91.2%, n = 31 ) highlighted the design approach undertaken to arrive at their robot prototype. Some studies employed a participatory design approach, in which prospective users were engaged in the conceptualisation, design, and prototype testing (Raj et al., 2023; Bouhali et al., 2023; Simao et al., 2018; Meghdari et al., 2018; Alves-Oliveira et al., 2022; Al Omoush et al., 2024; Maure and Bruno, 2023; Lupetti, 2017; Berrezueta-Guzman et al., 2021; Ruiz-del Solar et al., 2021). Other studies reported literature reviews as a crucial source of requirements and design features (Polyntsev et al., 2021; Yamini et al., 2024; Narejo et al., 2024; Baksh et al., 2024; Bouhali et al., 2023). Two studies, Polyntsev et al. (2021) and Yamini et al. (2024), give credit to their experience with existing robots as the source of their inspiration. Interestingly, animated movie characters were also mentioned to influence robot designs (Singh et al., 2023). The remainder (about 42%) described the robot development as primarily laboratory-based projects without external participatory input.

### 4.7 Demonstrated applications

We further examined the reviewed studies to determine whether the reported robotic systems had been deployed in real-world applications beyond laboratory settings. About 59% (n = 20) of the studies report field-based applications of their robots. As expected, due to our search query and study inclusion criteria, the majority of these field-based applications (70%, n = 14 ) were in the education domain (Alves-Oliveira et al., 2022; Shayan et al., 2016; Wu et al., 2016; Nagasawa and Yoshikubo, 2024; Galvez et al., 2022; Baksh et al., 2024; Budiharto, 2023; Maure and Bruno, 2023; Raj et al., 2023; Berrezueta-Guzman et al., 2021; Ivanova et al., 2025; Al Omoush et al., 2024; Jobaida et al., 2024; Lupetti, 2017). Other notable domains included health (Rodríguez-Calderón and Belmonte-Izquierdo, 2022; Ruiz-del Solar et al., 2021; Meghdari et al., 2018) and general interaction (Kim et al., 2024; Souza et al., 2024; Simut et al., 2016).

Further analysis of the robots deployed in educational settings (n = 14) revealed that about 43% (n = 6) interacted with children with special needs, including those with autism spectrum disorder (ASD) (Shayan et al., 2016; Wu et al., 2016; Ivanova et al., 2025), attention-deficit/hyperactivity disorder (ADHD) (Berrezueta-Guzman et al., 2021; Al Omoush et al., 2024), and children undergoing cancer treatment (Meghdari et al., 2018). In terms of instructional applications, six robots (43%) were deployed in the tutoring of topics related to STEM (Al Omoush et al., 2024; Galvez et al., 2022; Budiharto, 2023; Jobaida et al., 2024) and/or language learning (Shayan et al., 2016; Alves-Oliveira et al., 2022; Al Omoush et al., 2024; Jobaida et al., 2024).

### 4.8 Software and design availability

As the aim is to avoid unnecessarily re-inventing existing solutions, we analysed the availability of hardware and software of the robots. For hardware, a robot was considered fully available only if its body design, circuit, and overall architecture were either described in the article or provided through a supplementary file or public repository. For software, a robot was considered open source only when the authors explicitly stated this and provided access to a public repository.

Of the 34 robots, 26.5% (n = 9) provided complete hardware designs and also offered open-source software. Three robots (8.8%) offered neither hardware designs nor open-source software. Additionally, 29.4% (n = 10) had their hardware architecture described but used closed-source software. The remaining robots had mixed levels of hardware description and software availability. Table 7 summarises these findings.

**TABLE 7:** Hardware and software availability.

	Software availability		
Hardware described	Closed-source	Not indicated	Open-source
No	Souza et al. (2024), (Petrovič and Kostrian, 2024), Al Omoush et al. (2024)	Galvez et al. (2022), Simao et al. (2018), Nagasawa and Yoshikubo (2024), Raj et al. (2023)	Shayan et al. (2016)
Only the architecture	Ruiz-del Solar et al. (2021), Ma et al. (2020), Budiharto (2023), Kim et al. (2024), Zakipour et al. (2016), Meghdari et al. (2018), Diano and Claveau (2015), Bouhali et al. (2023), Simut et al. (2016), Jobaida et al. (2024)	Ivanova et al. (2025)	Polyntsev et al. (2021)
Only the body design		Wu et al. (2016)	
The architecture and the circuit design	Narejo et al. (2024), Malarvizhi et al. (2024)		
The architecture, circuit design, and body design	Lin (2022)		Rodríguez-Calderón and Belmonte-Izquierdo (2022), Maure and Bruno (2023), Berrezueta-Guzman et al. (2021), Sanchez-Orozco et al. (2025), Alves-Oliveira et al. (2022), Hayosh et al. (2020), Yamini et al. (2024), Baksh et al. (2024), Lupetti (2017)
Only the circuit design	Singh et al. (2023)		

### 4.9 Estimated prototype cost and associated analysis

As presented in Table 8, 53% (n = 18) of the studies explicitly report the total cost of their prototypes. Most of these studies also include a breakdown of the cost of the equipment used. About 72% (n = 13) of these robots cost less than USD 1000, with the cheapest reporting a total cost of USD 25.33. However, for the study by Nagasawa and Yoshikubo (2024) (S/N 11), it should be noted that the indicated cost does not include the acquisition of a smartphone.

**TABLE 8:** Robot cost.

S/N	Morphology description	Robot name	Total cost (USD)	Articles
1	Humanoid	Sanpo	25.33	Lin (2022)
2	Humanoid	Tiny	120.42	Jobaida et al. (2024)
3	An eye, nose, and mouth	Shybo	171	Lupetti (2017)
4	Plush animal	Probogotchi	174.31	Simut et al. (2016)
5	Humanoid	–	229.65	Al Omoush et al. (2024)
6	Cube-shaped with a screen, two flippers, and a base	Robotic Study Companion (RSC)	260	Baksh et al. (2024)
7	Head, torso, and wheels	Tinku	380	Singh et al. (2023)
8	Humanoid	Otisma	397	Bouhali et al. (2023)
9	A smartphone on a base with four mini legs	–	450	Diano and Claveau (2015)
10	Humanoid	Mirrly	499.57	Yamini et al. (2024)
11	Tabletop humanoid torso robot with dual gesture arms, projected 3D face, and smartphone head/interface	–	500	Nagasawa and Yoshikubo (2024)
12	Cat-like	Meow	670	Polyntsev et al. (2021)
13	Head, torso, and arms	Woody	718.68	Hayosh et al. (2020)
14	Head, torso, and a base	Flexi	2330.21	Alves-Oliveira et al. (2022)
15	Head, torso, and arms	Yaren	3650	Sanchez-Orozco et al. (2025)
16	Head, torso, arms, waist, and wheels	Arash	6000	Meghdari et al. (2018)
17	Head, torso, and arms	RASA	7000	Zakipour et al. (2016)
18	Humanoid	HBS-1	10000	Wu et al. (2016)
	**Median**		**474.8**	
	**IQR**		**1690.1**	
	**Mean**		**1865.3**	
	**Standard deviation** (σ)		**2903.2**	

Further analysis reveals that half of the robots cost less than USD 475, with the median value being USD 474.8. Nevertheless, the mean cost was USD 1865.3, which is considerably greater than the median, suggesting that a small number of comparatively expensive robots increased the overall average cost. Both the inter-quartile range (IQR) and standard deviation, USD 1690.1 and USD 2903.2, respectively, further highlight the variability in robot prices across the reviewed studies. In general, these findings suggest that many of the reviewed robots were developed at relatively lower costs despite the presence of some more expensive designs.

Additionally, we evaluated whether the robot morphology, compute, study area, and openness of software and/or hardware had any significant influence on the robot cost. Given the small sample size: only 18 robots had indicative total prototype costs, and even fewer with sufficient data for some feature-specific analysis, we opted for the Kruskal–Wallis test as it is a robust non-parametric test (alternative to one-way ANOVA) that does not assume normality, hence appropriate for small sample sizes and non-normally distributed data (Field et al., 2012). Consequently, epsilon-squared $\left({arepsilon}^{2}\right)$ was used to compute effect sizes. We also computed the median and IQR as they are more appropriate given the sample size. Table 9 details the results (note: all costs are in USD).

**TABLE 9:** Analysis of robot features and prototype cost.

Description	Frequency	Median	IQR	Mean	SD	H statistic	p-value	$\varepsilon^{2}$
Morphology								
Zoomorphic	2	422.16	247.84	422.16	350.51	2.50	0.29	0.04
Anthropomorphic (full body)	6	313.32	326.20	1878.66	3982.43			
Anthropomorphic (partial)	8	1524.44	3767.50	2593.74	2695.84			
Study area (Continent)								
Asia	7	397.00	3074.96	2038.91	3065.27	4.38	0.11	0.20
Europe	3	174.31	44.50	201.77	50.46			
North America	5	718.68	1830.64	2799.69	4099.00			
Main compute								
Arduino	2	98.16	72.83	98.16	103.00	7.43	0.06	0.32
Personal Computer	5	6000.00	4669.79	5100.90	3884.72			
SBC	9	397.00	410.00	769.48	1098.03			
Smartphone	2	475.00	25.00	475.00	35.36			
Software or hardware design availability								
Either is available (but not both)	3	670.00	4987.34	3565.11	5582.09	0.33	0.85	0.00
None is open source	9	397.00	270.35	1694.60	2738.78			
Both are open source	6	609.12	1607.44	1271.58	1408.11			

For the interpretation of effect sizes, we adopted commonly used benchmarks for $\varepsilon^{2}$ as highlighted by Iacobucci et al. (2023). No statistically significant associations were observed between cost and morphology ( p=0.29 ; $\varepsilon^{2}=0.04$ : small effect), study area ( p=0.11 ; $\varepsilon^{2}=0.20$ : moderate effect), choice of main compute ( p=0.06 ; $\varepsilon^{2}=0.32$ : large effect), and software or hardware design availability ( p=0.85 ; $\varepsilon^{2}=0.00$ : negligible effect). However, we stress that given the small sample size, these results are just exploratory and should be interpreted cautiously.

## 5 Discussion

This study set out to identify, through a systematic literature review, the features and capabilities commonly incorporated in low-cost social robots for educational contexts, as well as the design approaches and trade-offs that enable such capabilities while maintaining affordability. Unlike previous reviews that primarily synthesise educational outcomes or interaction paradigms, our analysis foregrounds affordability as a design constraint and examines how technical implementation choices shape the feasibility of deploying social robots at scale. This perspective is particularly relevant for low-resource educational contexts, where cost, manufacturability, and maintainability are as important as interaction quality.

This section further discusses these findings, highlighting their implications for design choices and practical considerations in low-resource contexts.

### 5.1 Robot appearance

Our findings indicate that designers have considerable creative freedom with respect to robot morphology. While the majority of the robots were anthropomorphic, zoomorphic and other non-anthropomorphic designs were also substantially represented. Moreover, among anthropomorphic robots, more than half were not fully humanoid. This suggests that, depending on the target demographic and specific characteristics of the intended users, researchers can innovatively determine the most appropriate robot morphology.

Although no statistically significant association was observed between robot morphology and cost, as shown in Table 8, all zoomorphic robots cost less than USD 700. Smartphone-based robots were generally priced around USD 500, excluding the smartphone itself, indicating that overall costs can be controlled through the choice of device. In the case of anthropomorphic robots, humanoid designs appeared at both the lower and higher ends of the cost spectrum, while partially anthropomorphic robots were similarly distributed. This indicates that cost variations are driven more by specific design features and components than by morphology alone. Consequently, researchers should not assume that any particular morphological category is inherently more expensive than another.

In addition, several noteworthy innovations related to robot housing were identified. With the increasing availability of 3D printing technologies, as also highlighted by Fang et al. (2026), many robot designs employ this approach to achieve low-cost yet visually appealing casings. The use of plush animals also emerged as an interesting option, as such materials are readily available and relatively inexpensive, even when custom-made. Moreover, plush-based housings can be reproduced for larger-scale deployment, while still allowing easy access to enclosed components for maintenance. Depending on contextual and pedagogical requirements, researchers can also create multiple variations of the same robot design to appeal to different learner groups. Collectively, the reviewed studies indicate that robot housing, although requiring careful and research-informed design, should not be a primary driver of cost escalation. Instead, the use of widely available, locally sourced materials is encouraged, both to minimise costs and to support scalability.

Another noteworthy observation concerns the predominance of white, brown, and wood-like colours in the reviewed robot designs. While it may be tempting to associate these preferences with the cultural or regional contexts in which the studies were conducted (see Figure 4), this is not explicitly discussed in the reviewed articles. Nevertheless, the predominance of white colour in robot designs is in line with the findings of Sparrow (2020), where they assessed whether robots have race. In their study, out of 125 “human-like” robots in the Anthropomorphic roBOT (ABOT) database, 72% were predominantly white, though some had other secondary colours. The discussion regarding robot colour matters because, as highlighted by Barenbrock et al. (2024), a robot’s surface colour can influence implicit stereotypical assumptions. More specifically, in a study by Barfield (2021), robot colour was directly associated with its perceived intelligence and trustworthiness, with the white-coloured robots being preferred as doctors and elementary school teachers. In educational settings, these assumptions may affect the learning process. As several studies report employing participatory design approaches and eliciting requirements from intended users, it is plausible that colour preferences may vary across contexts. Consequently, similar participatory approaches in different socio-cultural settings may yield different design preferences. The importance of such participatory approaches is also emphasised by Wei et al. (2025).

With regard to robot dimensions, the mean height (63.3 cm) indicates a preference for robots under 1 m tall. This likely reflects the fact that most of the reviewed robots were designed for child users, for whom smaller, less imposing embodiments are generally considered more appropriate and approachable (Podpečan, 2023). Additionally, larger robots are generally more difficult and costly to build.

### 5.2 Commonly incorporated features and components

When embarking on a robot design project, identifying the features a robot should possess is critical, as these choices directly influence both effectiveness and cost. In this context, it is useful to consider the qualities that define a “social robot,” as outlined by Mahdi et al. (2022), including sociability, social interaction, and social intelligence. In line with these qualities, our findings indicate that speech-based interaction is the most common feature, encompassing both speech recognition and synthesis, and enabling natural oral communication with learners. Vision capabilities were also frequently reported, supporting interaction through visual cues; enabling face, object, colour, and emotion recognition; as well as distance estimation for navigation. Notably, both speech and vision functionalities were often enabled through cloud-based or standalone software solutions, allowing these interaction capabilities to be implemented without substantially increasing the hardware cost of the robot prototype.

The ability to move body parts was also frequently reported as important. Interestingly, such movement was more often associated with non-verbal communication, which is key to interaction (Wolfert, 2024), rather than with the execution of manual tasks. Another feature identified as enhancing HRI was the ability to display content on a screen. This supports both non-verbal communication and the visualisation of supplementary learning materials, as in Pande and Mishra (2023), a common practice in tutoring robots. Mobility was reported in 23.5% of the reviewed studies and, depending on end-user requirements, may also be considered an important feature.

The aforementioned features were implemented using similar devices with varying specifications. The selection of sensors and actuators was largely guided by the specific capability requirements. This helped keep the robot costs manageable. An interesting practice was observed with regard to displays: some robots incorporated embedded LCD screens, others relied on attached smartphones, while a few projected content onto external displays. Therefore, depending on the intended use case and available resources, an appropriate display modality can be selected.

The conversion of smartphones into robots also emerged as an innovative and effective approach. It allows the utilisation of sensors, actuators, and applications readily available on the device. Moreover, additional applications can be developed and installed, or accessed remotely. Depending on their specifications, smartphones can provide adequate to substantial processing power to support a wide range of functionalities. Researchers should explore this option further, as smartphones are widely accessible (Theonest et al., 2024), making such solutions feasible and scalable even beyond school settings. Additionally, as most people are already familiar with operating smartphones (Theonest et al., 2024), the learning curve and maintenance burden are significantly reduced.

Furthermore, our findings shed light on the choice of compute for robot design, another crucial component that may affect cost. When the use case does not involve complex functionalities beyond the control of low-level sensors and actuators, a microcontroller (e.g., Arduino) is typically sufficient and represents a more cost-effective choice. Introducing an SBC (e.g., Raspberry Pi) in such cases may unnecessarily increase system cost and complexity. However, for applications requiring higher-level processing, such as AI-based functionalities, cloud connectivity, or graphical user interfaces (GUIs), an SBC becomes more appropriate. In many designs, a combined architecture can be adopted, where the microcontroller handles real-time control tasks while the SBC supports more computationally intensive operations.

### 5.3 Robot cost and affordability

Affordability is a relative concept, depending not only on available resources but also on what is considered reasonable expenditure. This is evident in the reviewed studies: robots such as Sanpo (USD 25.33) and HBS-1 (USD 10,000) were both described as low-cost by their developers. Nevertheless, the fact that nearly three-quarters of the reviewed robots cost less than USD 1,000 is promising for research laboratories in low-resource contexts. This aligns with the growing adoption of low-cost and open hardware technologies to improve access to scientific and educational equipment in resource-constrained settings (Wenzel, 2023; Maina et al., 2016). Depending on application needs, such costs may therefore be considered reasonably affordable.

### 5.4 Software and hardware design availability

Over 25% of the reviewed studies describe robots with open-source hardware and software, while most of the remaining studies provide varying levels of hardware design specifications and software availability. Only three studies included neither sufficient hardware design detail nor open-source software to enable meaningful technical comparison. This level of openness reduces barriers to entry in robot design and development. It also facilitates the advancement of robot features, as laboratories with varying expertise and resources can build upon and extend existing hardware and software.

### 5.5 Learner inclusion in the reviewed robots

Inclusion is an important consideration in the design of educational technologies, given the diverse needs of learners. Notably, about 26.5% (n = 9) of the reviewed robots explicitly targeted learners with special needs, including individuals with ASD, ADHD, those requiring sign language support, children with cancer, and refugee populations (Simao et al., 2018; Narejo et al., 2024; Singh et al., 2023; Ivanova et al., 2025; Bouhali et al., 2023; Simut et al., 2016; Meghdari et al., 2018; Zakipour et al., 2016; Berrezueta-Guzman et al., 2021). These robots employed a range of interaction capabilities, such as speech-based communication, expressive displays, body-part movements for non-verbal communication, salient social cues, and vision-based perception. In some cases, cloud-based services were utilised to support more advanced functionalities through AI-based models. While the present review does not categorise robots by target user groups, these findings indicate that the identified design features and capabilities are also applicable in specialised educational contexts. As such, researchers developing robots for learners with specific needs may draw on these design considerations when building effective low-cost systems.

### 5.6 Implications for researchers in low-resource contexts

The findings of this review may provide useful insights for researchers working in low- and middle-income contexts. While acknowledging that affordability is context-dependent, the reviewed robots are relatively less expensive than many commercially available alternatives, which may support greater accessibility. Moreover, this review outlines a range of design options and considerations that can support the development of custom robots using commercially available off-the-shelf components and locally available materials for robot housing. Although 3D printing technologies are increasingly available, their accessibility remains uneven in low- and middle-income contexts due to the cost of equipment and materials, maintenance, and infrastructure limitations, as also discussed by Olatunji et al. (2023). Consequently, practical alternatives, such as converting smartphones into robots or using plush animals and plywood for robot housing, may be more suitable in such settings.

In terms of design approach, some reviewed studies report the engagement of prospective users during the design phase as key to identifying specific requirements that guide design specifications and influence user acceptance. Where feasible, this participatory design approach may be beneficial, as specificity is central to achieving low-cost yet effective robot designs. This is particularly relevant in diverse educational contexts where design requirements may vary significantly.

Contextualising this further, for a country such as Tanzania, with over 18,000 public schools, additional innovation and creativity may be required if social robots are to be adopted and scaled within the education sector. Furthermore, while this study provides multiple pathways for achieving low-cost robot designs, broader contextual challenges must also be considered. These include reliable internet connectivity, stable electricity supply, and adequate budget allocation for connectivity and digital services, all of which are important considerations for sustainable deployment (Rutatola et al., 2025).

### 5.7 Future trends in low-cost social robotics for education

The cost of the reviewed robots with respect to their capabilities is promising. Trends show decreasing prices and increased availability of off-the-shelf hardware components that are required to build custom robots (Fezari et al., 2025; Balbuena et al., 2026). Moreover, the democratisation of 3D printing, which enhances customisation and production as also noted by Fang et al. (2026), and the innovative use of commonly available housing materials are also significant milestones. Additionally, the increased utilisation of cloud-based AI services, together with the growing availability of offline (local) models, suggests that advanced functionalities can be achieved without significantly increasing hardware requirements. Cloud services allow computation to be offloaded from the robot, while local models enable some level of autonomy on relatively low-cost hardware.

Therefore, these trends indicate a future where social robots are likely to become increasingly effective, accessible, and scalable, even in low-resource contexts and across diverse educational needs. As a result, a broader range of educational environments may become viable for deployment and experimentation, including longer-term deployments in the wild. This may significantly benefit the HRI research community by enabling richer and more diverse experiences from currently under-explored contexts.

## 6 Conclusions, limitations, and future work

Two research questions were addressed in this review. Regarding RQ1, the findings show that commonly incorporated robot features and capabilities include speech-based interactions, vision, movement of body parts, content and expressive displays, touch input, and, in some cases, mobility. Concerning RQ2, several approaches for ensuring that custom robots remain low-cost were identified. These include the use of creative and accessible housing designs such as 3D-printed casings; leveraging off-the-shelf sensors, actuators, and computing components aligned with the intended requirements; and externally incorporating robotic functionalities into smartphones to benefit from their built-in compute, sensors, and actuators. Collectively, these findings demonstrate that even researchers in low-resource contexts can design affordable custom robots suitable for research and real-world applications.

Admittedly, this study has several limitations. First, the focus on systematically retrievable published works may have led to the omission of relevant robot designs that are not documented in scholarly literature. In particular, the search was limited to four major academic databases (Web of Science, Scopus, IEEE Xplore, and ACM Digital Library) and did not include grey literature or additional databases, which may have excluded some relevant designs. Nevertheless, this limitation is mitigated by the methodological rigour and depth of analysis applied to the retrieved studies, enabling robust and well-supported insights within the scope of the review. Second, restricting the search to educational contexts may have excluded innovative robot designs developed for other domains. However, the reported findings are presented in a manner that allows them to inform robot design decisions beyond educational settings. Additionally, inclusion required that studies provide sufficient technical detail on the robot’s hardware and software design within the paper itself. As a result, studies that primarily reported the use of existing robots, referred to other publications for key design details, or provided limited technical descriptions were excluded, which may have led to the omission of relevant platforms used in educational contexts. Lastly, although this study focused on robot designs reported in peer-reviewed publications, the included studies provide varying levels of detail regarding hardware and software specifications, which may have constrained the depth of comparison in some cases.

While this review provides detailed guidance for designing affordable custom robots, several areas warrant further empirical investigation. Future research could, for example, examine children’s preferences regarding robot morphology (anthropomorphic *versus* zoomorphic) and housing materials (e.g., plastic *versus* plush), and how these factors influence learning outcomes and engagement. Additionally, although custom robots are often shown to be effective, further comparative studies examining their durability, maintainability, and long-term resilience relative to commercial robots would be valuable. In a similar vein, regardless of the robot type, it is also important to explore associated costs to robot adoption, which could include connectivity, software subscriptions, teacher training, electricity, and technical support. This is crucial as the overall robot cost extends beyond initial development or acquisition.

## Data Availability

The original contributions presented in the study are included in the article/supplementary material, further inquiries can be directed to the corresponding author.

## Appendix 1: The search queries used in each database

### Appendix A : Web of Science

TS=((“social robot*” OR “humanoid robot*” OR “anthropomorph*” OR “human-like robot*”)

AND (“design*” OR “feature*” OR “appearance” OR “embodiment”)

AND (education* OR classroom OR teach* OR learner* OR tutor* OR peer)

AND (affordab* OR “low-cost” OR “cost-effective*” OR “open source” OR “open-source” OR “open hardware” OR “DIY” OR “Arduino” OR “Raspberry Pi”)

AND robot*

NOT (“industrial robot*” OR “manufacturing robot*”))

AND PY=(2015–2025)

### Appendix B : Scopus

TITLE-ABS-KEY((“social robot*” OR “humanoid robot*” OR “anthropomorph*” OR “human-like robot*”)

AND (“design*” OR “feature*” OR “appearance” OR “embodiment”)

AND (education* OR classroom OR teach* OR learner* OR tutor* OR peer)

AND (affordab* OR “low-cost” OR “cost-effective*” OR “open source” OR “open-source” OR “open hardware” OR “DIY” OR “Arduino” OR “Raspberry Pi”)

AND robot*

AND NOT (“industrial robot*” OR “manufacturing robot*”))

AND PUBYEAR > 2014 AND PUBYEAR < 2026.

### Appendix C : The IEEE Xplore

(“All Metadata”:“social robot*” OR “All Metadata”:“humanoid robot*” OR “All Metadata”:“anthropomorph*” OR “All Metadata”:“human-like robot*”)

AND (“All Metadata”:“design*” OR “All Metadata”:“feature*” OR “All Metadata”:“appearance” OR “All Metadata”:“embodiment”)

AND (“All Metadata”:education* OR “All Metadata”:classroom OR “All Metadata”:teach* OR “All Metadata”:learner* OR “All Metadata”:tutor* OR “All Metadata”:peer)

AND (“All Metadata”:affordab* OR “All Metadata”:“low-cost” OR “All Metadata”:“cost-effective*” OR “All Metadata”:“open source” OR “All Metadata”:“open-source” OR “All Metadata”:“open hardware” OR “All Metadata”:“DIY” OR “All Metadata”:“Arduino” OR “All Metadata”:“Raspberry Pi”)

AND (“All Metadata”:robot*)

NOT (“All Metadata”:“industrial robot*” OR “All Metadata”:“manufacturing robot*”)

Note: The publication year (2015–2025) was filtered afterwards.

### Appendix D : The ACM Digital Library

[[Abstract: “social robot*”] OR [Abstract: “humanoid robot*”] OR [Abstract: “anthropomorph*”] OR [Abstract: “human-like robot*”]]

AND [[Abstract: “design*”] OR [Abstract: “feature*”] OR [Abstract: “appearance”] OR [Abstract: “embodiment”]]

AND [[Abstract: education*] OR [Abstract: classroom] OR [Abstract: teach*] OR [Abstract: learner*] OR [Abstract: tutor*] OR [Abstract: peer]]

AND [[Abstract: affordab*] OR [Abstract: “low-cost”] OR [Abstract: “cost-effective*”] OR [Abstract: “open source”] OR [Abstract: “open-source”] OR [Abstract: “open hardware”] OR [Abstract: “diy”] OR [Abstract: “arduino”] OR [Abstract: “raspberry pi”]]

AND [Abstract: robot*]

AND NOT [[All: “industrial robot*”] OR [All: “manufacturing robot*”]]

AND [E-Publication Date: (01/01/2015 TO 12/31/2025)]

Note: This was built on the ACM query string builder.
